# Draft Genome Sequence of *Hymenobacter* sp. Strain IS2118, Isolated from a Freshwater Lake in Schirmacher Oasis, Antarctica, Reveals Diverse Genes for Adaptation to Cold Ecosystems

**DOI:** 10.1128/genomeA.00739-14

**Published:** 2014-08-07

**Authors:** Hyunmin Koo, Travis Ptacek, Michael Crowley, Ashit K. Swain, John D. Osborne, Asim K. Bej, Dale T. Andersen

**Affiliations:** a Department of Biology, University of Alabama at Birmingham, Birmingham, Alabama, USA; b Department of Microbiology, University of Alabama at Birmingham, Birmingham, Alabama, USA; c Heflin Center for Genomic Sciences, University of Alabama at Birmingham, Birmingham, Alabama, USA; d Geological Survey of India, Antarctic Division, Faridabad, India; e Carl Sagan Center, SETI Institute, Mountain View, California, USA

## Abstract

*Hymenobacter* sp. IS2118, isolated from a freshwater lake in Schirmacher Oasis, Antarctica, produces extracellular polymeric substance (EPS) and manifests tolerance to cold, UV radiation (UVR), and oxidative stress. We report the 5.26-Mb draft genome of strain IS2118, which will help us to understand its adaptation and survival mechanisms in Antarctic extreme ecosystems.

## GENOME ANNOUNCEMENT

*Hymenobacter* sp. IS2118, isolated from a seasonally ice-covered freshwater lake (L43) in Schirmacher Oasis, Antarctica, is a psychrotolerant, Gram-negative, red-pigmented bacterium that thrives in the Antarctic environment, which is extremely cold and dry and has high levels of solar UV radiation (UVR) (1). *Hymenobacter* sp. have been previously reported to manifest various metabolic capabilities pertaining to their resistance to oxidative stress (2), such as production of copious amounts of extracellular polymeric substance (EPS) (3) and synthesis of unique UVR-protective 2′-hydroxy-carotenoid pigments (4), which have potential applications in biotechnology and biomedicine (5, 6).

We describe here a draft genome of *Hymenobacter* IS2118 to elucidate the key metabolic and stress-tolerance genes relating to the survival of these organisms in extreme environments. The genomic DNA from IS2118 cultures was extracted using a MoBIO PowerSoil DNA purification kit. The genome was sequenced on an Illumina Miseq instrument (250-bp paired-end reads), producing 5,420,359 reads. The adapter sequences were checked by FastQC (http://www.bioinformatics.babraham.ac.uk/projects/fastqc/) and then trimmed by Trimmomatic (7). The trimmed sequences were *de novo* assembled using ABySS 1.3.6 (8). After iterative testing of k-mer lengths, we selected an assembly (k-mer length, 155) with 199 contigs with sizes ranging from 162 bp to 151,115 bp (total length, 5,262,580 bp). GC content was 60.7%, with a read depth of 553×.

We annotated the assembled genome using Rapid Annotation using Subsystem Technology (RAST) (9). We used RNAmmer (10), tRNAscan-SE (11), and ARAGORN (12) databases and found 57 predicted RNAs, including 1 rRNA operon, 45 tRNAs, 1 transfer-messenger RNA (tmRNA), and 10 miscellaneous RNAs. The RAST result showed 4,821 protein-coding genes (coding sequences [CDSs]), including 1,360 known and 3,461 unknown subsystems.

We also found 63 stress-responsive genes, including 36 in oxidative, 7 in osmotic, 6 in periplasmic stress, 4 in cold shock (3 *cspA* and 1 *cspG*), and 10 in detoxification categories. Additionally, we identified 131 genes within the cell wall and capsule category, including 44 associated with EPS, 27 associated with Gram-negative cell wall components, and 60 under no subcategory. Also, we found 54 genes for isoprenoid pigment biosynthesis (11 carotenoids) and 51 genes related to DNA repair (UvrABC system, *recA*, and *uvrD*), 18 genes related to multidrug resistance (MDR) efflux pump activities (ABC transporter, multidrug and toxin extrusion [MATE] family of MDR, and macrolide-specific *macA* efflux pump), 10 prophages (3 phage tails, 3 replications, and 4 lysis proteins), 9 restriction-modification systems, 1 YcfH DNase, 3 outer membrane nucleases, 1 secondary metabolite (phenylpropanoid apigenin derivative), and 1 pathogenic island close to the *Listeria* LIPI-1 gene cluster, as well as genes for the persister cell phenotype (*hipA* and *sulA*). Interestingly, the IS2118 genome harbors 3 teichoic and lipoteichoic acid biosynthesis genes, which are characteristically found in Gram-positive bacteria and were previously reported to have cryoprotective roles in subzero temperature environments (13). Additionally, secondary metabolites were found through antiSMASH (14), giving a 7-gene cluster (4 bacteriocin, 2 terpene, and 1 mixed terpene/polyketide synthase). The genome of IS2118 revealed a suite of diverse stress-responsive and pigment-producing genes, along with genes typically found in Gram-positive bacteria, which will enable us to better understand the survival mechanisms of this bacterium in cold ecosystems and its importance in biotechnology and biomedicine.

### Nucleotide sequence accession numbers.

This whole-genome shotgun project has been deposited in DDBJ/ENA/GenBank under the accession no. JNLX00000000. The version described in this paper is the first version, JNLX01000000.
